# The relationship of sleep duration and quality to cognitive impairment in cART‐treated HIV positive adults aged 50 and older in Kilimanjaro, Tanzania

**DOI:** 10.1002/alz.085062

**Published:** 2025-01-09

**Authors:** Megan Patricia Sison, Sarah Urasa, Marieke Dekker, Jane Mosha, Zawadi Minde, Sean Davidson, Lachlan Fotheringham, Aloyce Kisoli, Miranda Worth, Molly Sharpe, Bethany Burrus, Richard Walker, Stella‐Maria Paddick

**Affiliations:** ^1^ Newcastle University, Newcastle, Newcastle Upon Tyne United Kingdom; ^2^ Kilimanjaro Christian Medical University College, Moshi Tanzania, United Republic of; ^3^ Cumbria, Northumberland, Tyne and Wear NHS Foundation Trust, Newcastle United Kingdom; ^4^ Northumbria Healthcare NHS Foundation Trust, North Shields United Kingdom

## Abstract

**Background:**

HIV‐associated neurocognitive disorders (HAND) are prevalent complications of ageing with treated HIV, disproportionally affecting sub‐Saharan Africa. Causal HAND treatments are lacking worldwide; therefore, reversible factors are important to explore. Sleep duration and quality are frequently associated with risk of cognitive impairments. We explored the relationship between sleep duration and quality with cognitive impairments in older people living with HIV (PLWH) in Tanzania.

**Method:**

This cross‐sectional observational study involved systematically sampled participants aged ≥50 undergoing longitudinal follow‐up for HAND in Kilimanjaro, Tanzania. Sleep duration and quality were measured subjectively using sleep diaries, the Chalder Fatigue Questionnaire and Epworth Sleepiness Scale, and objectively by wrist‐worn accelerometers. Contextual and subjective factors affecting sleep were evaluated using a locally‐developed questionnaire. HAND was diagnosed using 2007 ‘Frascati’ consensus criteria based on neuropsychological tests, clinical assessments, and informant histories. HIV disease severity was assessed using blood markers and case‐note review.

**Result:**

Among 103 participants, 41 met criteria for HAND whilst 12 had non‐HAND cognitive impairments. Fragmented sleep (58.3%) and short nocturnal sleep durations (53.4%) were most prevalent. Average total sleep time (6.4 hours) was below the recommended seven hours. Every one‐point increase in Chalder Fatigue Questionnaire score increased the risk of HAND with functional impairment and non‐HAND cognitive impairments by 11.6% *(Odds ratio = 1.116, CI(1.012,1.231))*. Epworth Sleepiness Scale score and total sleep time were not associated with cognitive impairment.

**Conclusion:**

These pilot data suggest significant association between symptomatic HAND and non‐HAND cognitive impairments with fatigue in older PLWH. Future studies should examine longitudinal risk and relationship with clinically‐diagnosed sleep disorders.

